# PREMATURE DEATH ATTRIBUTABLE TO SOCIAL DETERMINANTS OF HEALTH AMONG ASIAN AMERICANS: A POPULATION-BASED COHORT

**DOI:** 10.1093/geroni/igae098.0377

**Published:** 2024-12-31

**Authors:** Xiang Qi, Katherine Wang, Bei Wu

**Affiliations:** New York University, New York, New York, United States; Duke University, Durham, North Carolina, United States; New York University, New York, New York, United States

## Abstract

Asian adults are one of the fastest growing ethnic minority groups in the US and have higher levels of unfavorable social determinants of health (SDoH), including limited English fluency, unemployment, and reduced healthcare access, yet the impact of these SDoH on mortality remains understudied in this population. This study aims to examine the prospective associations of multiple SDoH with premature all-cause mortality, and to investigate their respective contribution among a nationally-representative cohort of Asian Americans, using data from 3,888 Asian adults aged 20-74 who participated in the National Health and Nutrition Examination Survey 2011-2018. Cox proportional hazards regression models were employed to examine the relationships of SDoH factors with mortality; and population attributable fractions (PAFs) were calculated to assess the contributions of each individual SDoH on premature death. We found that unemployment, limited English proficiency, social isolation, no health insurance, in poverty, housing instability, less education, and food insecurity were significantly associated with mortality (HRs range from 1.20 to 2.35). SDoH explain up to 38.6% of mortality in the Asian Americans, among them, unemployment (18.5%), limited English fluency (9.0%), and social isolation (8.3%) showed the highest relative contributions to mortality. These results challenge the “Model Minority” stereotype, often perpetuated by English-only national survey data, and highlight the unique SDoH challenges faced by Asian Americans. Future research disentangling SDOHs on health outcomes by Asian subgroup is necessary to dispel the “model minority” stereotype that remains prevalent and provide better informed preventive practices and interventions for this population.

